# Impact of late gadolinium enhancement extent, location, and pattern on ventricular tachycardia and major adverse cardiac events in patients with ischemic vs. non-ischemic cardiomyopathy

**DOI:** 10.3389/fcvm.2022.1026215

**Published:** 2022-10-18

**Authors:** Emily Tat, Caroline Ball, Gerald P. Camren, Igor Wroblewski, Khaled A. Dajani, Ari Goldberg, Menhel Kinno, Thriveni Sanagala, Mushabbar A. Syed, David J. Wilber, Mark Rabbat

**Affiliations:** ^1^Department of Internal Medicine, Columbia University Medical Center, New York, NY, United States; ^2^Division of Cardiology, Loyola University Medical Center, Maywood, IL, United States; ^3^Department of Radiology, Loyola University Medical Center, Maywood, IL, United States

**Keywords:** late gadolinium enhancement, cardiac magnetic resonance, ischemic cardiomyopathy, non-ischemic cardiomyopathy, ventricular tachycardia, major adverse cardiac events

## Abstract

**Background:**

Left ventricular late gadolinium enhancement (LGE) by cardiac magnetic resonance (CMR) has been associated with increased risk for life-threatening ventricular tachyarrhythmias. The differences in association between LGE characteristics and prognosis in patients with ischemic (ICM) vs. non-ischemic (NICM) cardiomyopathy is incompletely understood.

**Methods:**

A total of 168 consecutive patients who underwent CMR imaging with either ICM or NICM were included in our study. LGE extent, location and pattern were examined for association to the primary endpoint of ventricular tachycardia (VT) and secondary endpoint of major adverse cardiac events (MACE).

**Results:**

Of 68 (41%) patients with ICM and 97 (59%) patients with NICM, median LGE mass was 15% (IQR 9–28) for the ICM group and 10% (IQR 6–15) for the NICM group. On multivariate analysis for both groups, LGE characteristics were prognostic while LVEF was not. In patients with ICM, septal and apical segment LGE, and involvement of multiple walls predicted both endpoints on multivariate analysis. LGE extent (≥median) and inferior wall LGE independently predicted the primary endpoint. In patients with NICM, anterior, inferior and apical segment LGE, and involvement of multiple walls predicted both endpoints on multivariate analysis. LGE extent (≥median, number of LGE segments, LGE stratified per 5% increase) and midwall LGE were independent predictors of the primary endpoint.

**Conclusions:**

Although LGE was an independent predictor of prognosis in both groups, LGE extent, location, and pattern characteristics were more powerful correlates to worse outcomes in patients with NICM than ICM.

## Introduction

The placement of cardiovascular implantable electronic devices (CIED) are important for the prevention of sudden cardiac death in patients at high risk for life-threatening ventricular tachyarrhythmias. While significant benefit has been demonstrated in patients with ischemic cardiomyopathy (ICM), the use of device therapy in patients with non-ischemic cardiomyopathy (NICM) is controversial (1–4). The DANISH trial demonstrated conflicting outcomes for the use of prophylactic ICD implantation in patients with NICM (5). Other studies have found benefit for ICD implantation in reducing morbidity and mortality in these patients (6, 7). Despite low LVEF as a primary indication for device therapy, many patients with LVEF > 35% experience sudden cardiac death (8, 9). There are likely additional contributory factors that increase risk for sudden cardiac death.

Recent advances in cardiac magnetic resonance (CMR) have expanded our ability to non-invasively evaluate cardiac structure, function and perform tissue characterization (10). With the incorporation of gadolinium-based contrast agents, areas of perfusion defects, myocardial necrosis and scar can be identified and quantified (11). Further, late gadolinium enhancement (LGE) patterns can differentiate ischemic vs. non-ischemic forms of myocardial injury. Myocardial fibrosis, assessed by LGE on CMR, has been proposed to have prognostic value for ventricular tachyarrhythmias and sudden cardiac death in patients with ICM and NICM (12–15). The majority of data on the impact of LGE on prognosis is limited to LGE presence and extent. Further, the relationship of other LGE characteristics such as location and pattern are incompletely understood.

Amongst NICM, CMR tissue characterization techniques, such as LGE, T1/T2 mapping, ECV, and T2 assessment, have been established in diagnosis, risk stratification, and guidance of management (16). In patients with NICM, CMR can identify patients at increased risk for sudden cardiac death but who are not included in current guidelines for primary prevention implantable cardioverter-defibrillators (17), such as those with midwall LGE and LV ejection fraction ≥40% (18, 19). CMR can identify key structural findings in both ischemic cardiomyopathy and non-ischemic cardiomyopathy (20, 21) to identify the best candidates for implantable cardioverter-defibrillators (22). The aim of this study was to evaluate the differences in prognostic relevance of LGE extent, pattern and location in patients with ICM vs. NICM to predict long-term outcomes in a real-life cohort.

## Methods

### Study population and design

A total of 349 consecutive patients from May 1, 2013 to December 12, 2016 who underwent CMR for cardiomyopathy evaluation at Loyola University Medical Center (Maywood, IL) were identified. Data was retrieved for all adult patients (>18 years) with either ICM or NICM as confirmed on CMR. All patients without contraindication to CMR examination with use of gadolinium contrast were included. In addition, the diagnosis of congenital heart disease as the etiology of their cardiomyopathy and presence of both ICM and NICM precluded their inclusion to the study. Patients with inadequate tissue characterization on CMR due to CIED interference were also excluded. Using this criteria, a total of 168 consecutive cases were included in our study.

### CMR imaging

All CMR imaging was performed using a Siemens Trio 3-T or Siemens Aera 1.5-T scanner (Siemens Medical Solutions, Malvern, Pennsylvania, USA) using our institution's standardized CMR protocol. In brief, CMR images were acquired using retrospective ECG gated steady-state free precession cine sequence (slice thickness 7 mm, slice gap 3 mm, echo time 1.2 ms, repetition time 47.1 ms, matrix 208 × 180, and field of view 300 mm). Short axis slices covering the entire left ventricle were acquired at end-expiration. The border of the left atrium and left ventricle was delineated by the atrioventricular plane with more than 50% of the circumference surrounded by left ventricular myocardial tissue. End-diastole was the visually estimated image stack with the largest left ventricular cavity. End-systole was defined as the smallest left ventricular cavity by visual estimation. Delayed enhancement imaging was performed 10 min after an intravenous injection of gadolinium (0.1–0.2 mmol/kg; gadoterate dimeglumine). Two readers evaluated LGE localization and quantification and LGE quantification was performed using the full width at half maximum method of short axis slices through the left ventricle using commercially available software. Inter-observer reliability was calculated using a random sample of 10 patients (5 ICM, 5 NICM) and found to be 0.78 for the ICM cohort and 0.91 for the NICM cohort. The average measure intraclass correlation was 0.86 (CI −0.39–0.99, *p* = 0.044) for the ICM cohort, and 0.85 (CI −0.46–0.98, *p* = 0.048) for the NICM cohort. All CMR images were analyzed using Circle Cardiovascular Imaging (Circle Cardiovascular Imaging, Inc, Calgary, Alberta, Canada). Chamber quantification and wall motion assessment were performed by the interpreting physician.

### CMR characteristics

**Figure 1:**
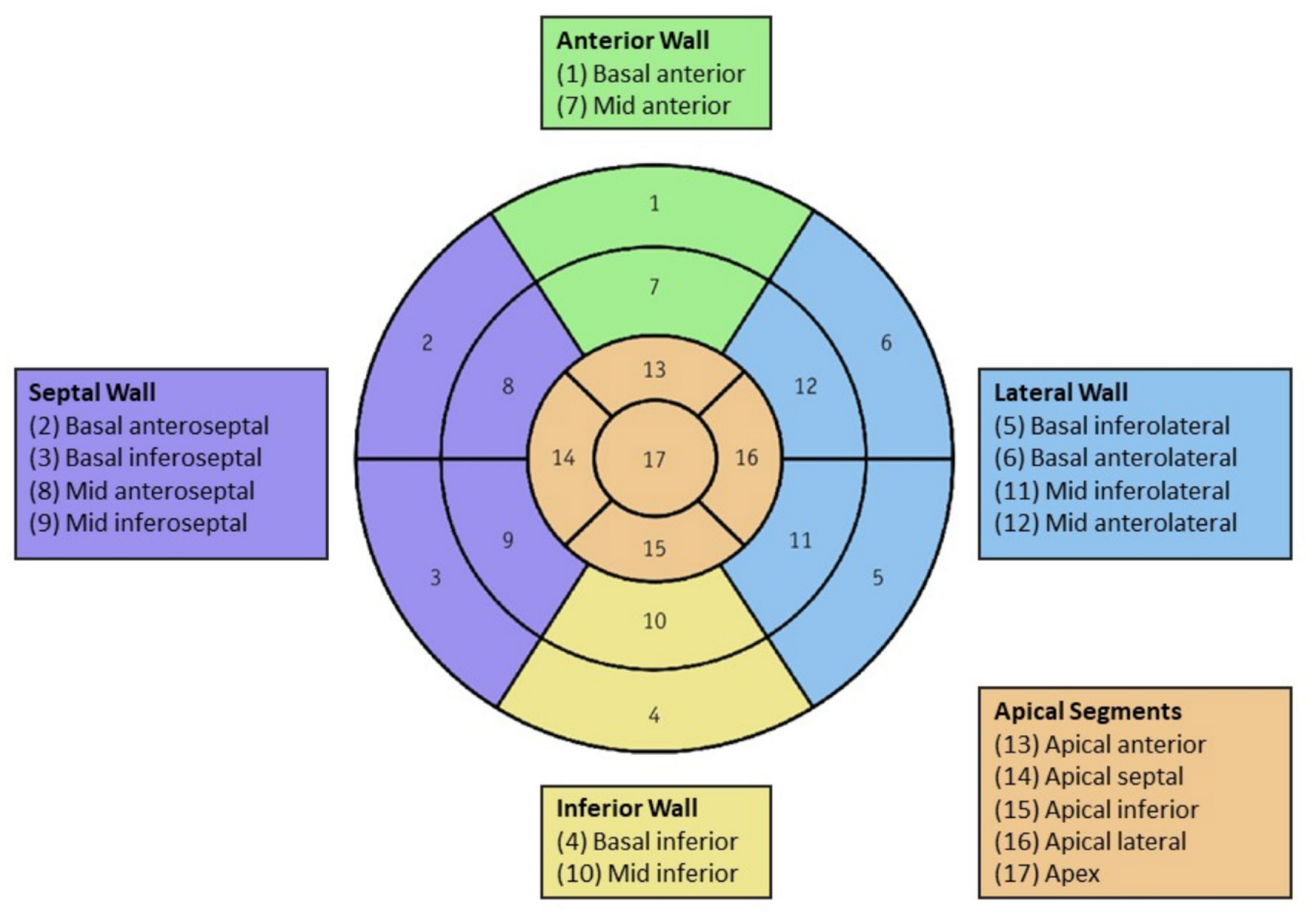
Left ventricular segmentation: The 17-segment left ventricle model as defined by the American Heart Association distributed into five larger segments: anterior (1, 7), lateral (5, 6, 11, 12), inferior (4, 10), septal (2, 3, 8, 9), and apical (13–17). Color should be used for Figures.

Patients were classified into ICM or NICM groups based on scar pattern on the CMR. The following CMR characteristics were collected for all patients: left ventricular end-diastolic volume index (LVEDV), left ventricular end-systolic volume index (LVESV) and LVEF. LGE extent was quantified by percent, LGE greater than the median of respective ICM and NICM groups, per 5% increase, and by number of involved segments. The percent of LGE was calculated by dividing the LGE mass by the LV mass indexed to body surface area, then multiplying the quotient by 100. This percent range was further stratified by 5% increments to yield four groups: >0 and <5, ≥5 and <10, ≥10 and <15, and ≥15%. The 17-segment model as defined by the American Heart Association was utilized for number of involved segments with LGE out of 17, and number of involved locations with LGE (0–5) (23). From this model, the LV myocardium was divided into five larger segments: anterior (1, 7), lateral (5, 6, 11, 12), inferior (4, 10), septal (2, 3, 8, 9), and apical (13–17) for data analysis (Figure 1). LGE pattern was stratified by cardiomyopathy: ICM pattern was defined as either subendocardial or transmural, and NICM pattern was defined as either subepicardial or mid-wall.

### Clinical characteristics

Data on clinical characteristics was collected using the institution's electronic medical records. This included demographics, comorbidities, NYHA functional class, and medications.

### Endpoints

The primary endpoint was episode of ventricular tachycardia (VT) either sustained or non-sustained, defined as an episode lasting <30 s. The secondary endpoint was major adverse cardiac events (MACE) as defined as a composite of all-cause mortality, sudden cardiac death, non-fatal cardiac arrest, hospitalization for heart failure, and sustained ventricular tachycardia. Death and readmission data were determined through review of our institution's electronic medical record. Arrhythmia data was obtained through both review of the patient's clinical record and through device interrogation reports for patients with CIED. Non-fatal cardiac arrest included any malignant arrhythmia (sustained ventricular tachycardia or ventricular fibrillation), pulseless electrical activity (PEA), or asystole that the patient survived. Sudden cardiac death was defined as sudden death from a malignant arrhythmia, PEA, or asystole. If patients had more than one event occur (i.e., hospital admission for heart failure and later, episode of VT), each event was recorded.

### Statistical analysis

Categorical variables were summarized as numbers and percentages. Continuous variables were summarized as mean ± standard deviation (SD), skewed variables were presented as median (interquartile range). Comparisons by cardiomyopathy (ICM vs. NICM) were made using Independent-Samples *T*-tests for categorical variables with normal distribution, or Mann-Whitney *U*-tests for non-normal distribution. Continuous variables were compared using Pearson's Chi-Square analysis. Univariate and multivariate Cox regression analysis were performed to determine predictors of primary and secondary endpoints using hazard ratio (HR) and 95% confidence intervals (CI). Reliability analysis was performed for inter and intra-observer variability assessment. A 2-tailed *p* ≤ 0.05 was considered statistically significant. Statistical analysis was performed using IBM SPSS Statistics Version 23.0 (IBM Corp., Armonk, New York). The study protocol was approved by our institutional review board.

## Results

### Baseline characteristics

A total of 168 consecutive patients with either ICM or NICM who underwent CMR were included in our study. Baseline clinical characteristics are outlined in Table 1. The mean age of the cohort was 57 ± 15 years with 43 (26%) females. A total of 97 (59%) patients were identified to have NICM and 68 (41%) patients with ICM. The median NYHA Class of the cohort was NYHA class II. Patients with NICM were more often female (32 vs. 18%, *p* = 0.039) and younger (52 ± 17 vs. 63 ± 10 years, *p* < 0.001) than patients with ICM. Patients with ICM had more comorbidities of hypertension (71 vs. 47%, *p* = 0.003), hyperlipidemia (75 vs. 44%, *p* < 0.001), diabetes mellitus (41 vs. 21%, *p* = 0.004) and were more often smokers (38 vs. 20%, *p* = 0.008). ICM patients were more likely to be NYHA Class III or IV compared to NICM patients (35 vs. 14%, *p* = 0.017). In patients with ICM, 10 (7%) patients had obstructed coronaries of the left main, 52 (76%) of the left anterior descending, 38 (55%) of the left circumflex, 40 (58%) of the right coronary artery. Of this patient subgroup, 36 (53%) underwent a PCI and 35 (51%) had a CABG. In patients with NICM, the etiologies included myocarditis (12, 12%), tachy-mediated CM (11, 11%), sarcoidosis (9, 9%), LV non-compaction (7, 7%), chemotherapy-induced (4, 4%), takotsubo CM (3, 3%), postpartum CM (2, 2%), amyloidosis (1, 1%), thyrotoxicosis (1, 1%), alcohol-induced (1, 1%), and valvular (1, 1%).

**Table 1:** Baseline characteristics of the cohort.

**Characteristics**	**Total (*n* = 165)**	**ICM (*n* = 68)**	**NICM (*n* = 97)**	***P*-value**
Age (years)	57 ± 15	63 ± 10	52 ± 17	<0.001
Gender, female	43 (26)	12 (18)	31 (32)	0.04
Body mass index (kg/m^2^)	29 ± 7	28.6 ± 6	29.5 ± 7	0.37
Smoking	45 (27)	26 (38)	19 (20)	0.01
Hypertension	94 (57)	48 (71)	46 (47)	0.003
Hyperlipidemia	94 (57)	51 (75)	43 (44)	<0.001
Diabetes mellitus	48 (29)	28 (41)	20 (21)	0.004
NYHA functional class	1.9 ± 0.8	2.1 ± 1.0	1.8 ± 0.7	0.04
NYHA functional class				
I	55 (33)	21 (31)	34 (35)	
II	72 (44)	23 (34)	49 (51)	0.02
III	33 (20)	20 (29)	13 (13)	
IV	5 (3)	4 (6)	1 (1)	
Medications				
ACE or ARB	110 (67)	44 (65)	66 (68)	0.74
Calcium channel blocker	15 (9)	4 (6)	11 (11)	0.28
Diuretic	85 (52)	34 (50)	51 (53)	0.75
Nitrate	14 (9)	10 (15)	4 (4)	0.02
Beta blocker	129 (78)	57 (84)	72 (74)	0.18
Antithrombotic agent	109 (66)	57 (84)	52 (54)	<0.001
Statin	93 (56)	60 (88)	33 (34)	<0.001
Amiodarone	23 (14)	13 (19)	10 (10)	0.12

Values are presented as mean ± standard deviation or number (percentage).

### CMR characteristics

CMR characteristics are outlined in Table 2. Volumetric quantification yielded a mean LVEDV index of 130 (101–150) ml/m^2^ and LVESV index of 82 (55–113) ml/m^2^. Mean LVEF was 34 ± 12 % with 85 (52%) patients with LVEF <35%. There was no statistical difference in LVEDV, LVESV, or LVEF between patients with ICM and NICM. The ICM group had greater LGE burden with 15% (9–28) LGE of the LV mass vs. 10% (6–15) LGE in the NICM group, *p* < 0.001. In addition, LGE was present in a median of 6 (4–8) segments within a 17-segment model of the left ventricle compared to 2 (0–3) segments in the NICM group (*p* < 0.001). LGE location was also significantly greater in patients with ICM across the anterior, lateral, inferior, septal walls and apical segments (*p* < 0.001). LGE pattern along a subendocardial distribution was observed in 27 (42%) of the ICM group with a median of 4 (1–6) segments involved in the 17-segment model. A transmural LGE pattern was observed in 38 (59%) patients with a median of 2 (0–5) segments. Within the NICM group, a subepicardial pattern was observed in 12 (12%) patients with a median of 1 (0–2) segment involved. More often, a mid-wall pattern was observed (28 patients, 29%) with the involvement of 2 segments (0–3).

**Table 2:** CMR characteristics of the cohort.

**Characteristics**	**Total (*n* = 165)**	**ICM (*n* = 68)**	**NICM (*n* = 97)**	***P*-value**
LV EDV index (ml/m^2^)	130 (101–150)	122 (105–150)	130 (102–153)	0.75
LV ESV index (ml/m^2^)	82 (55–113)	86 (63–113)	78 (49–113)	0.17
LV EF (%)	34 ± 12	32 ± 11	35 ± 13	0.09
LV EF ≤ 35%	85 (52)	39 (47)	46 (57)	0.21
**LGE extent**				
LGE mass (gr)	6.7 (4–11)	8.7 (6–16)	5.5 (3–8)	0.046
LGE mass (% of LV)	11 (7–17)	15 (9–28)	10 (6–15)	<0.001
LGE >0, <5%	17 (12)	1 (2)	16 (19)	0.002
LGE ≥5, <10%	39 (27)	16 (28)	23 (27)	0.91
LGE ≥10, <15%	32 (22)	10 (17)	22 (26)	0.24
LGE ≥15%	48 (33)	30 (52)	18 (21)	<0.001
Number of LGE segments (0–17)	3 (0–6)	6 (4–8)	2 (0–3)	<0.001
**LGE location**				
Anterior wall	40 (24)	31 (46)	9 (9)	<0.001
Lateral wall	63 (38)	38 (56)	25 (26)	<0.001
Inferior wall	57 (34)	36 (53)	21 (22)	<0.001
Septal wall	76 (46)	48 (72)	28 (29)	<0.001
Apical segments	56 (34)	46 (69)	10 (10)	<0.001
Number of LGE location (0–5)	2 (2–3)	3 (2–4)	1 (0–2)	<0.001
**LGE pattern**		***n*** **(%)**	**Segments**	***n*** **(%)**	**Segments**	
Subendocardial				27 (42)	4 (1–6)		
Transmural				38 (59)	2 (0–5)		
Subepicardial		12 (12)	1 (0–2)	
Mid wall		28 (29)	2 (0–3)	

Values are presented as mean ± standard deviation, median (interquartile range) or number (percentage).

### Long-term outcomes

Long-term outcomes of the cohort are outlined in Table 3. Mean time for follow-up was 23 ± 14 months, with no statistical difference between ICM or NICM groups (24 ± 13 vs. 23 ± 15 months, *p* = 0.641). Our primary endpoint of at least one episode of VT either sustained or non-sustained occurred in 53 (32%) of the cohort. Our secondary endpoint of all-cause mortality, sudden cardiac death, non-fatal cardiac arrest, hospitalization for heart failure, and sustained VT defined as MACE was met in 63 (38%) patients. During the observation period, hospitalization for heart failure occurred in 43 (26%) patients. Non-fatal cardiac arrest occurred in 13 (8%) patients. Sudden cardiac death occurred in 2 (1%) patients. All-cause mortality was met in 23 (14%) patients, of which included 12 cardiac-related deaths and 11 non-cardiac related deaths. There was no difference in outcomes for MACE, hospitalization for heart failure, VT, or all-cause mortality between the two groups.

**Table 3:** Long-term outcomes of the cohort.

**Characteristics**	**Total (*n* = 165)**	**ICM (*n* = 68)**	**NICM (*n* = 97)**	***P*-value**
CIED implantation	76	32 (47)	44 (45)	0.52
**Primary endpoint**				
VT sustained or non-sustained	53 (32)	24 (35)	29 (30)	0.47
VT sustained	27 (16)	13 (19)	14 (14)	0.42
VT non-sustained	42 (26)	18 (27)	24 (25)	0.80
**Secondary endpoint**				
MACE	63 (38)	28 (41)	35 (36)	0.51
All-cause mortality	23 (14)	13 (19)	10 (10)	0.12
Sudden cardiac death	2 (1)	2 (3)	0 (0)	0.09
Non-fatal cardiac arrest	13 (8)	7 (10)	6 (6)	0.34
Hospitalization for heart failure	43 (26)	14 (21)	29 (30)	0.18

Values are presented as number (percentage).

### Predictors of primary and secondary outcomes

#### LGE predictors of outcomes for the entire cohort

The results from univariate Cox regression analyses for predictors of primary and secondary endpoints are summarized in Table 4. Type of cardiomyopathy (ICM or NICM) was not predictive of VT (HR 0.87, CI 0.47–1.62, *p* = 0.66) or MACE (HR 1.02, CI 0.57–1.84, *p* = 0.94). LVEF ≤ 35% was not predictive of the primary endpoint (*p* = 0.12) but was associated with risk for MACE (HR 2.80, CI 1.47–5.36, *p* = 0.002). LGE mass was predictive of both outcomes (HR 1.06, CI 1.02–1.10, *p* = 0.004 for VT; HR 1.04, HR 1.01–1.08, *p* = 0.02 for MACE). Percent of LGE was strongly predictive of VT (HR 14.14, CI 4.03–19.61, *p* = 0.048) but not MACE (*p* = 0.46). LGE burden by number of segments was also predictive of VT (HR 1.09, CI 1.02–1.18, *p* = 0.02 but not MACE (*p* = 0.10). Additionally, presence of LGE at the lateral wall (HR 2.00, CI 1.08–3.73, *p* = 0.03) and the involvement of multiple walls (HR 1.20, CI 1.02–1.44, *p* = 0.048) was associated with VT but not MACE (*p* = 0.118, *p* = 0.13, respectively).

**Table 4:** Predictors of primary and secondary endpoints in the entire cohort with univariate Cox regression analysis.

	**VT**			**MACE**		
	**HR**	**95% CI**	***P*-value**	**HR**	**95% CI**	***P*-value**
Age (years)	1.01	0.99–1.03	0.32	0.99	0.97–1.01	0.22
BMI	1.01	0.97–1.06	0.52	1.03	0.99–1.07	0.11
Etiology	0.87	0.47–1.62	0.66	1.02	0.57–1.84	0.94
**CMR**						
LV EDV index (ml/m^2^)	0.98	0.95–1.00	0.09	1.01	1.01–1.02	<0.001
LV ESV index (ml/m^2^)	1.66	0.88–3.15	0.12	1.01	1.01–1.02	<0.001
LV EF (%)	0.98	0.95–1.00	0.09	0.95	0.93–0.98	<0.001
LV EF ≤ 35%	1.66	0.88–3.15	0.12	2.80	1.47–5.36	0.002
**LGE extent**						
LGE mass (gr)	1.06	1.02–1.10	0.004	1.04	1.01–1.08	0.02
LGE mass (% of LV)	14.14	4.03–19.61	0.05	2.54	0.80–25.59	0.46
LGE >0, <5%	1.17	0.41–3.29	0.77	0.42	0.10–1.72	0.23
LGE ≥5, <10%	0.49	0.20–1.16	0.10	0.58	0.28–1.21	0.15
LGE ≥10, <15%	0.80	0.36–1.82	0.60	1.76	0.91–3.36	0.09
LGE ≥ 15%	1.42	0.74–2.73	0.29	1.58	0.87–2.89	0.14
Number of LGE seg	1.09	1.02–1.18	0.02	1.05	0.99–1.11	0.10
**LGE location**						
Anterior wall	1.31	0.76–2.58	0.44	1.03	0.64–1.96	0.93
Lateral wall	2.00	1.08–3.73	0.03	1.59	0.89–2.84	0.12
Inferior wall	1.73	0.93–3.3	0.08	1.59	0.89–2.84	0.12
Septal wall	1.11	0.59–2.08	0.74	1.21	0.68–2.15	0.51
Apical segments	1.50	0.80–2.8	0.21	1.27	0.71–2.27	0.43
Number of LGE locations (0–5)	1.20	1.02–1.44	0.04	1.13	0.96–1.34	0.13

#### LGE predictors of outcomes for patients with ICM

Multivariate analysis identified LGE extent and location as independent predictors for worse outcomes when adjusted for age, sex and body mass index (BMI) (Table 5). Specifically, LGE % mass greater than the median of 15% was associated with risk for VT (HR 1.28, CI 1.03–1.99, *p* = 0.049) and not MACE (p=0.68). LGE extent when stratified per 5% increase in LGE and number of involved segments were not associated with primary or secondary endpoints. Presence of LGE at the septal wall was the strongest predictor for VT (HR 5.96, CI 3.38–8.67, *p* = 0.02) and MACE (HR 4.05, CI 3.81–10.37, *p* = 0.04). Presence of LGE on multiple walls correlated to both outcomes (HR 1.31, CI 1.13–1.67, *p* = 0.01 for VT; HR 1.17, CI 1.13–1.95, *p* = 0.04 for MACE). LGE involving the apical segments and inferior wall were also predictive of outcomes. Left main coronary artery occlusion was associated with MACE (HR 4.7, CI 1.65–13.53, *p* = 0.004) but not VT (HR 0.82, CI 0.18–3.66, *p* = 0.79). Left anterior descending, left circumflex, right coronary artery involvement was not associated with either endpoints. LGE pattern and LVEF were not predictive of either primary or secondary outcomes.

**Table 5:** Predictors for primary and secondary outcomes with patients with ICM on multivariate analysis adjusted for age, sex, and BMI.

	**VT**			**MACE**		
	**HR**	**95% CI**	***P*-value**	**HR**	**95% CI**	***P*-value**
LV EF ≤ 35%	1.62	0.40–6.55	0.50	0.40	0.10–1.54	0.18
**LGE extent**						
LGE mass ≥ 15%	1.28	1.03–1.99	0.049	1.28	0.40–4.13	0.68
LGE >0, <5%	NS			NS		
LGE ≥5, <10%	1.54	0.72–4.94	0.96	1.64	0.23–11.68	0.62
LGE ≥10, <15%	1.34	0.91–8.58	0.51	1.28	0.12–13.96	0.84
LGE ≥15%	1.58	0.81–11.88	0.65	1.04	0.16–6.68	0.97
Number of LGE segments (0–17)	1.13	0.81–1.57	0.47	1.19	0.91–1.56	0.20
**LGE location**						
Anterior wall	1.20	1.03–1.56	0.13	1.36	1.05–2.65	0.32
Lateral wall	1.14	1.02–1.36	0.08	0.92	0.85–3.75	0.44
Inferior wall	1.12	1.02–2.00	0.05	1.12	1.01–1.33	0.84
Septal wall	5.96	3.38–8.67	0.02	4.05	3.81–10.37	0.04
Apical segments	1.08	1.01–2.83	0.03	1.06	1.00–1.96	0.05
Number of LGE locations (0–5)	1.31	1.13–1.67	0.01	1.17	1.13–1.95	0.04
**LGE pattern**						
Subendocardial	0.98	0.83–2.63	0.48	1.50	0.99–1.77	0.28
Transmural	1.13	0.90–2.73	0.19	0.99	0.60–3.80	0.93
Both	1.09	0.23–5.11	0.92	2.83	0.75–10.67	0.13
**Coronary artery occlusion**						
Left main	0.82	0.18–3.66	0.79	4.7	1.65–13.53	0.004
Left anterior descending	0.61	0.18–2.01	0.41	0.66	0.23–1.90	0.45
Left circumflex	0.44	0.13–1.46	0.18	1.19	0.49–2.91	0.70
Right coronary artery	1.63	0.41–6.42	0.49	0.49	0.17–1.40	0.18

#### LGE predictors of outcomes for patients with NICM

In patients with NICM, LGE extent, location, and pattern were independent predictors for worse outcomes on multivariate analysis when adjusted for age, sex and BMI (Table 6). Anterior and inferior wall, apical segment involvement, and involvement of multiple walls were identified as predictors of primary and secondary endpoints. Anterior wall LGE was the strongest predictor of VT (HR 60.81, CI 5.14–80.34, *p* = 0.001) while also strongly associated with MACE (HR 7.04, CI 1.54–14.58, *p* = 0.045). LGE involvement of multiple walls was the strongest predictor of MACE (HR 30.12, CI 3.76–45.30, *p* = 0.005) and also prognostic for VT (HR 30.12, CI 4.09–40.93, *p* = 0.01). LGE extent by all three definitions was predictive of VT but not MACE. Specifically, LGE mass greater than the median of 10% was associated with VT (HR 1.20, CI 1.09–2.51, *p* = 0.01). When stratified per 5% increase of LGE mass, only LGE>15% was significantly associated with risk for VT (HR 32.35, CI 3.16–45.24, *p* = 0.003) and not MACE (p=0.18). In addition, number of segments with LGE was associated with VT (HR 2.50, CI 1.07–5.84, *p* = 0.03) and not MACE (p=0.83). Midwall LGE was strongly associated with the primary endpoint (HR 10.22, CI 1.46–17.56, *p* = 0.02) where subepicardial LGE or presence of both were not. LVEF was not associated with either primary or secondary outcomes.

**Table 6:** Predictors for primary and secondary outcomes with patients with NICM on multivariate analysis adjusted for age, sex, and BMI.

	**VT**			**MACE**		
	**HR**	**95% CI**	***P*-value**	**HR**	**95% CI**	***P*-value**
LV EF ≤ 35%	0.74	0.42–1.29	0.29	0.68	0.42–1.09	0.11
**LGE extent**						
LGE mass ≥ 10%	1.20	1.09–2.51	0.006	2.38	0.90–6.29	0.08
LGE >0, <5%	1.43	0.25–8.20	0.98	0.73	0.12–4.31	0.73
LGE ≥5, <10%	1.76	0.90–3.12	0.51	0.86	0.13–2.45	0.44
LGE ≥10, <15%	2.00	0.92–2.58	0.13	1.34	0.74–5.33	0.69
LGE ≥15%	32.35	3.16–45.24	0.003	1.36	0.77–2.63	0.18
Number of LGE segments (0–17)	2.50	1.07–5.84	0.03	1.00	0.71–1.31	0.83
**LGE location**						
Anterior wall	60.81	5.14–80.34	0.001	7.04	1.54–14.58	0.04
Lateral wall	1.56	0.73–3.98	0.56	3.76	0.77–9.26	0.21
Inferior wall	13.55	2.64–17.14	0.02	5.97	3.40–20.45	0.02
Septal wall	2.37	0.90–14.19	0.35	4.33	0.48–30.86	0.19
Apical segments	27.73	3.55–36.93	0.02	5.44	2.21–20.55	0.03
Number of LGE locations (0–5)	30.12	4.09–40.93	0.01	30.12	3.76–45.30	0.005
**LGE pattern**						
Subepicardial	6.49	0.76–10.51	0.09	0.79	0.06–9.84	0.86
Mid wall	10.22	1.46–17.56	0.02	0.92	0.16–5.33	0.93
Both	1.03	0.34–3.13	0.95	1.34	0.38–4.17	0.08

## Discussion

This study demonstrated several findings: (1) The impact of LGE characteristics varied in ischemic vs. Non-ischemic cohorts, with greater impact of LGE extent, location, and pattern in patients with NICM than ICM (2) In patients with ICM, LGE extent, location (septal, inferior, apical segment LGE) and involvement of multiple LV walls were independent predictors of worse outcomes, while LGE pattern was not prognostic. (3) In patients with NICM, LGE extent, location (anterior, inferior and apical segment LGE), involvement of multiple LV walls, and mid-wall LGE pattern yielded prognostic significance for the prediction of worse outcomes. (4) LVEF≤35% was not prognostic in either ICM or NICM groups. Together, these observations are consistent with the hypothesis that LGE based scar has important prognostic utility in identifying a subset of patients that may benefit from ICD implantation. Specifically, the results from our study suggests that LGE extent, distribution, location, and pattern may lend prognostic utility for the creation of a risk-stratification model.

The replacement of functional myocardium with scar tissue is widely accepted as the substrate for which reentry-driven arrhythmias can occur (24). Thus far, numerous studies have found LGE-based scar to be a better predictor for life-threatening ventricular tachyarrhythmias than LVEF (25, 26). Our results are consistent with these findings: LGE was an independent predictor of primary and secondary endpoints of VT and MACE, respectively, while LVEF was non-contributory. While the impact of LGE presence has been well-reported, the association of LGE extent, location and pattern to adverse outcomes is limited. To our knowledge, the impact of these characteristics has not yet been defined between ICM and NICM groups. Our study adds to the literature by reporting differences in LGE characteristics between the two groups.

### LGE extent

Previous studies have found LGE to correlate to worse outcomes in patients with ICM (12, 27) and NICM cohorts (13, 28, 29). The relationship between LGE extent and increased risk for VT may represent an increased susceptibility to reentrant ventricular arrhythmias. For our study, LGE extent was evaluated by median of the cohort (30, 31), per incremental increase (28), and by number of segments (27) as utilized in previous studies. LGE extent was consistently significant using these three definitions in our cohort of NICM patients but using only one definition (LGE ≥ median) in ICM patients.

In addition, while patients with ICM tended to have higher LGE burden than patients with NICM, LGE extent was found to be a stronger predictor of outcomes in the NICM group compared to the ICM group. A meta-analysis by Disertori et al. similarly found the association of LGE to prognosis to be more impactful in patients with NICM with an odds ratio of 6.27 (CI 4.15–9.47) compared to ICM with an odds ratio of 5.05 (CI 2.73–9.36) (12). Given the additional comorbidities and risk for increased structural remodeling post-myocardial infarction that occurs in patients with ICM, LGE may likely be part of a multifactorial process for what ultimately drives these outcomes in this cohort.

In patients with NICM, the impact of LGE extent in the literature, while overall strongly correlative to worse outcomes, is highly variable. In our study, LGE greater than the median of 10% and >15% when stratified per 5% increase were significantly associated with risk for VT. LGE cut-offs in the literature found to have prognostic value have been reported from just 1, 5% (29), 8% (31) to 13% (28). The variability in these findings is likely a reflection of different patient populations, methodology for LGE quantification, and most importantly, the heterogenous composition of the various cardiomyopathies that make up NICM. Hypertrophic cardiomyopathy, cardiac amyloid, genetic dilated cardiomyopathy, cardiac sarcoidosis, and anthracycline cardiomyopathy are several of many etiologies that are individually distinct in LGE extent, location and pattern (32). The heterogenous results in the literature for NICM may be a reflection of the heterogenous composition that makes up each NICM cohort.

### LGE location

In our study, LGE location was the greatest predictor of primary and secondary endpoints in both groups on multivariate analysis that included consideration to LGE extent, pattern, and LVEF. The role of LGE location and involvement of multiple walls in the left ventricle, therefore, may be as equally prognostic, if not more so, than LGE extent in both ICM and NICM patient populations. In a study of 874 NICM patients by Halliday et al. predictive models using LGE presence and location performed better than models using LGE extent and pattern (29).

In patients with ICM, septal wall LGE was the strongest overall predictor of worse outcomes. This finding is similar to the literature for myocarditis, in which septal LGE was associated with worse prognosis in these patients (33). Likely, involvement of the conduction system with attenuation to the right ventricle are likely contributory (28). While septal wall LGE was not prognostic in our NICM cohort, our findings of lateral and inferior wall LGE have been previously demonstrated (28). In our cohort, anterior wall LGE was the overall strongest predictor of VT. The underlying mechanism for how LGE location correlates to worse outcomes is not yet understood. Certainly, further studies with attention to LGE location are required. An important finding in our study was the correlation of multiple wall involvement to increased risk for VT and MACE in both NICM and ICM groups. This finding, combined with the impact of LGE location, suggests that the distribution of scar tissue is key to driving aberrant conduction, as opposed to high burden LGE. These results are consistent with our current understanding of reentrant mechanisms, in which heterogenous zones are created at the periphery of scar tissue, from which a flux of functional and non-functional myocardium allows the propagation of abnormal electrical activity (6).

### LGE pattern

The relationship between LGE pattern and prognosis has been inconsistently reported. In patients with ICM, while a transmural LGE pattern trended toward an increase in VT, and presence of both subendocardial and transmural pattern trended toward increased risk for MACE, these findings were not significant on univariate and multivariate analysis. There is currently limited literature on the effects of LGE pattern to risk for arrhythmia in patients with ICM.

In patients with NICM, sub-epicardial patterns have been reported to be associated with sudden cardiac death, while midwall patterns were found to be associated with mortality (22). Barison et al. found no significant association between LGE pattern and risk for ICD shock or a composite of ICD shock and cardiac death (28). In our study, sub-epicardial LGE trended toward an increased risk for VT but was not found to be significant. LGE with a midwall pattern, however, was strongly associated with VT and not MACE. Similarly, a previous study found midwall LGE to be associated with a 9-fold increased risk for sudden cardiac death in patients with dilated cardiomyopathy and LVEF > 40% (18). Given that reentry formation requires the presence of viable myocardium adjacent to scar tissue as previously mentioned, presence of midwall fibrosis may serve as the substrate for adjacent subepicardial and subendocardial arrhythmias to propagate.

Consistent with these findings, the creation of a risk stratification system, ideally based on artificial intelligence taking into consideration the potential for bias, should take a much more personalized approach instead of sole reliance on LVEF or LGE presence (34). Type of cardiomyopathy, from ischemic vs. non-ischemic, with consideration of the specific type of non-ischemic cardiomyopathy, should be used in congruence with LGE characteristics of extent, pattern, and location to best identify patients that would benefit from CIED therapy.

## Study limitations

This is a single center retrospective study with a limited sample size. While the procedure is streamlined within our institution, there is currently no consensus or standardized procedure to quantify LGE. In addition, LGE-CMR diagnosis and interpretation are operator dependent. Our study did not utilize more advanced CMR methods such as extracellular volume fraction or parametric mapping. Such studies may provide a more accurate representation of abnormal myocardial substrate, especially for diffuse fibrosis. Certainly, prospective, large-volume studies are warranted to best confirm our findings to better explain the differences in LGE impact on patients with ICM vs. NICM. Specifically, for patients with NICM, the impact of LGE extent, location, and pattern correlated with outcomes for specific types of NICM are lacking and would benefit this cohort significantly. Additionally, the evaluation of NICM and LGE does not account for the etiology of NICM which may impact the prognosis of this patient population. Regarding our endpoint of ventricular tachycardia, we acknowledge that while this can be detected in patients with a CIED, asymptomatic VT may be missed in patients without a CIED. Finally, we did not analyze peri-infarct (or “gray zone”) volumes.

## Conclusion

Presence of myocardial scar detected by LGE-CMR has potential to help risk stratify which patients are at highest risk for life-threatening ventricular tachyarrhythmias. LGE was an independent predictor of worse outcomes in patients with ICM and NICM. However, LGE characteristics were more prognostic for NICM patients with attention to extent, location, and pattern than ICM patients, and may likely yield more utility in their ability to risk stratify this cohort. In addition, LGE location and the distribution of scar may be more powerful markers for mortality than LGE extent. In summary, our findings suggest that the future utility of LGE may serve to replace LVEF and to augment the approach to CIED therapy strategies through characterization and personalization of LGE characteristics.

## Data availability statement

The original contributions presented in the study are included in the article/supplementary material, further inquiries can be directed to the corresponding author.

## Author contributions

ET: conceptualization, formal analysis, data curation, writing—original draft, and visualization. CB: conceptualization, methodology, verification, data curation, and writing—review and editing. GC, KD, AG, MK, TS, MS, and DW: writing—review and editing and validation. IW: data curation and writing—review and editing. MR: conceptualization, methodology, validation, writing—review and editing, and supervision. All authors contributed to the article and approved the submitted version.

## Conflict of interest

The authors declare that the research was conducted in the absence of any commercial or financial relationships that could be construed as a potential conflict of interest.

## Publisher's note

All claims expressed in this article are solely those of the authors and do not necessarily represent those of their affiliated organizations, or those of the publisher, the editors and the reviewers. Any product that may be evaluated in this article, or claim that may be made by its manufacturer, is not guaranteed or endorsed by the publisher.

## Abbreviations

CIED: cardiovascular implantable electronic devices
LVEF: left ventricular ejection fraction
NYHA: New York Heart Association
ICM: ischemic cardiomyopathy
NICM: non-ischemic cardiomyopathy
CMR: cardiac magnetic resonance
LGE: late gadolinium enhancement
LVEDV: left ventricular end-diastolic volume
LVESV: left ventricular end-systolic volume
VT: ventricular tachycardia
MACE: major adverse cardiac events
SD: standard deviation
HR: hazard ratio
CI: confidence intervals
BMI: body mass index.
